# Immunohistochemistry for Thymidine Kinase-1 (TK1): A Potential Tool for the Prognostic Stratification of Breast Cancer Patients

**DOI:** 10.3390/jcm10225416

**Published:** 2021-11-19

**Authors:** Giuseppe Nicolò Fanelli, Rosa Scarpitta, Paola Cinacchi, Beatrice Fuochi, Anna Szumera-Ciećkiewicz, Katia De Ieso, Paola Ferrari, Andrea Fontana, Mario Miccoli, Antonio Giuseppe Naccarato, Cristian Scatena

**Affiliations:** 1 Division of Pathology, Department of Translational Research and New Technologies in Medicine and Surgery, University of Pisa, 56126 Pisa, Italy; giuseppenicolo.fanelli@phd.unipi.it (G.N.F.); rosa.scarpitta@for.unipi.it (R.S.); b.fuochi@studenti.unipi.it (B.F.); giuseppe.naccarato@unipi.it (A.G.N.); 2 Department of Laboratory Medicine, Pisa University Hospital, 56126 Pisa, Italy; ka.deieso@ao-pisa.toscana.it; 3 Unit of Oncology 1, Department of Medical and Oncological Area, Pisa University Hospital, 56126 Pisa, Italy; p.cinacchi1@studenti.unipi.it (P.C.); p.ferrari@ao-pisa.toscana.it (P.F.); 4 Unit of Oncology 2, Department of Medical and Oncological Area, Pisa University Hospital, 56126 Pisa, Italy; an.fontana@ao-pisa.toscana.it; 5 Department of Pathology and Laboratory Diagnostics, Maria Sklodowska-Curie National Research Institute of Oncology, 02-781 Warsaw, Poland; anna.szumera-cieckiewicz@pib-nio.pl; 6 Department of Diagnostic Hematology, Institute of Hematology and Transfusion Medicine, 02-776 Warsaw, Poland; 7 Department of Clinical and Experimental Medicine, University of Pisa, 56126 Pisa, Italy; mario.miccoli@unipi.it

**Keywords:** breast cancer, thymidine kinase-1, prognosis, distant recurrence-free survival, overall survival, chemotherapy

## Abstract

Breast cancer (BC) is the most frequent non-cutaneous malignancy in women. Histological grade, expression of estrogen and progesterone receptors (ER and PgR), overexpression/amplification of the human epidermal growth factor receptor 2 (HER2) oncogene, and proliferative activity measured with ki-67 provide important information on the biological features of BC and guide treatment choices. However, a biomarker that allows a more accurate prognostic stratification is still lacking. Thymidine kinase-1 (TK1), a ubiquitous enzyme involved in the pyrimidine nucleotide recovery pathway, is a cell-proliferation marker with potential prognostic and predictive impacts in BC. Eighty (80) cases of invasive BC with a long-term follow-up were retrospectively selected, and clinicopathological data were collected for each patient. TK1 tissue expression was evaluated immunohistochemically. Data suggested that TK1 expression levels are positively correlated with ER and PgR expression, and negatively correlated with HER2 status and the impact on patients’ distant recurrence-free survival (DRFS): in detail, among patients undergoing adjuvant chemotherapy, lower TK1 levels are correlated with better DRFS. Therefore, these results contribute to furthering the knowledge of TK1, suggesting a possible and important role of this enzyme as a biomarker in the stratification of BC patients.

## 1. Introduction

Breast cancer (BC) represents the most frequent non-cutaneous malignant tumor in women, accounting for approximately 30% of all cancer diagnoses [1]. Its incidence varies according to age: very low before the age of 25, increasing rapidly after the age of 30, peaking around perimenopause, and gradually decreasing in the elderly [2]. In addition, BC also represents the second leading cause of cancer death in females worldwide, after lung cancer [1]. In the last 20 years, gene expression studies have highlighted a considerable heterogeneity within BC, leading to the identification of subtypes with differences in biological characteristics and clinical course [3,4]; some of these, such as triple-negative cancers (TNBC), have a poor prognosis and limited therapeutic possibilities [5]. In clinical practice, histological grade, expression of estrogen and progesterone receptors (ER and PgR), overexpression of the human epidermal growth factor receptor 2 (HER2) oncogene, and proliferative activity, measured by ki-67, represent crucial parameters that provide important information on the biology of BC, which then guide the choice of treatment. However, despite all the efforts to control the disease, a considerable number of patients experience relapse and metastasis, resulting in a very poor prognosis [6]. Therefore, the actual risk stratification in BC is only partially defined, and new biomarkers that identify patients with a more aggressive disease are urgently needed. Widely studied over the past 20 years, thymidine kinase-1 (TK1) is a ubiquitous enzyme that plays a key role in the pyrimidine nucleotide recovery pathway for DNA damage synthesis and repair [7,8]. TK1, like ki-67, is a cell proliferation marker, and is probably even more accurate due to its close association with the S phase of the cell cycle [9,10,11,12,13]. Growing evidence also points to its central role as a proliferation marker in BC [12]: indeed, high levels of TK1 are found in BC tumors with elevated proliferative activity [14,15] and are associated with aggressive characteristics of the tumor (advanced stage, high grade, ER/PgR negativity, tumor necrosis, vascular invasion) [16]. These findings potentially make TK1 a valuable tool for identifying patients at risk of disease recurrence [17]. The aim of this work was to correlate the expression of TK1 in tumoral specimens of BC with patients’ prognosis, particularly in terms of distant recurrence-free survival (DRFS), in order to define a cut-off-value to be used in routine practice for the stratification of the risk of relapse and, consequently, tailor the best management individually for each patient.

## 2. Materials and Methods

### 2.1. Case Selection

A total of 80 BC patients were retrospectively selected from the archives of the Pathology Unit of the University Hospital of Pisa (from 2004 to 2016). Rigorous inclusion criteria have been applied: (i) a long-term follow-up (at least 10 years); (ii) no neoadjuvant chemotherapy administration; (iii) selection of surgical specimens from the primary tumor (patients with only BC biopsy were excluded); (iv) pathological confirmation (biopsy or surgical specimens) of distant recurrence; (v) adequate and sufficient tissue of the primary BC for the tissue microarray (TMAs) creation (see below).

Patients were well-balanced among two groups: (i) “metastatic/relapsed”, composed of 40 patients who experienced recurrence or synchronous/metachronous distant metastases; (ii) “non-metastatic/relapsed”, which included 40 patients who did not relapse until the end of the observation period (30 November 2020). Among all patients, 18 had a multicentric/multifocal BC: 10 in the “metastatic/relapsed” group and 8 in the “non-metastatic/relapsed” group.

For all cases, the following information was annotated: patient’s age at primary BC diagnosis; cancer histotype (according to the 2019 World Health Organization—WHO—classification) [18]; TNM and stage (according to the 2017 American Joint Cancer Committee—AJCC—Cancer Staging Manual) [19]; grade (according to Nottingham combined histologic grade) [20]; invasive cancer size (histologically measured as the largest diameter); the number of metastatic lymph nodes; ER and PgR status (annotating the percentage of their expression) [21]; HER2 overexpression/amplification (confirmed with dual-probe in situ hybridization—DISH) [22]; proliferation index ki-67 [23]; and surrogated molecular subtype (according to ER, PgR, and HER2 status, as well as proliferation index) [24].

All tissue samples were processed according to standard protocols [25]. All cases were jointly reassessed by three pathologists with expertise in breast cancer (G.N.F., C.S. and A.G.N.), and representative formalin-fixed and paraffin-embedded (FFPE) blocks were selected for the construction of the TMAs.

From the medical records, the administration of adjuvant chemotherapy, the distant recurrence, and the cancer-related death times was annotated. Only patients with a pathological confirmation (biopsy or surgical specimens) of distant recurrence were included (see inclusion criteria above). Distant recurrence-free survival (DRFS) was defined as the time from surgery to the time of first distant recurrence. Overall survival (OS) was defined as the time from the primary BC diagnosis to the patient’s cancer-related death. Living patients were censored at the date of the last follow-up.

All investigations were conducted according to the principles expressed in the Declaration of Helsinki; this study was approved by the Ethical Committee for the testing and evaluation of clinical study protocols in the North “Wester Tuscany Area (CEAVNO)” (Prot. n: 17770. Approval date: 9 July 2020). Written informed consent was obtained from each participant.

### 2.2. TMA Construction

Three areas of invasive BC and one area of normal breast tissue were selected and marked on hematoxylin- and eosin-stained sections of each sample. These areas were transferred onto FFPE tissue blocks, and the coordinates were recorded. Tissue cores (2 mm in diameter) were punched out of these areas using the TMA Grand Master (3DHISTECH, Budapest, Hungary), according to the manufacturer’s instructions. Each tissue core was tidily embedded in the recipient paraffin blocks according to the grid designed with the TMA Control software (3DHISTECH), which corresponded to a TMA map (spreadsheet) indicating the position and identification of each core. In each TMA block, liver, tonsil, kidney, testis, and thyroid tissue cores were used as controls. Finally, 297 tissue cores of primitive BC and 99 tissue cores of normal tissue were collected in 10 TMA blocks (amounting to 396 cores overall).

### 2.3. Immunohistochemistry (IHC)

One 4 µm-thick section from each TMA block was cut. Slides were incubated with rabbit monoclonal anti-TK1 antibody (clone: EPR3193) (Abcam, Cambridge, UK, dilution 1:100) at 40 °C for 60 min (Ventana Benchmark XT staining system, Ventana Medical System—Roche) and developed in diaminobenzidine (DAB)–hydrogen peroxide for 10 min (ultraView Universal DAB kit, Ventana Medical System—Roche). Finally, sections were counterstained with hematoxylin and mounted.

Positivity for TK1 was calculated as the percentage of tumor cells (or normal ductal epithelial cells) with cytoplasmic expression of TK1, regardless of the intensity. Each core was evaluated independently, and the mean expression of TK1 was calculated among the cores of each tumor. All cores were evaluated blindly by three pathologists with expertise in breast cancer (C.S., G.N.F. and A.G.N.); discrepancies were resolved via consensus on a multi-head microscope.

### 2.4. Statistical Analysis

**Figure A1:**
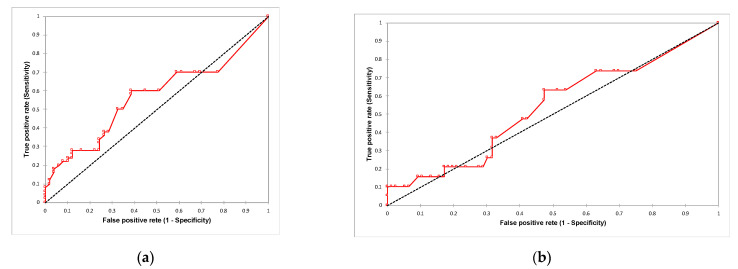
ROC curve analyses: (**a**) the AUC value is 0.57 for the DRFS (*p =* 0.244); (**b**) and 0.54 for the OS (*p =* 0.642); black dashed line: random chance; red line: ROC curve.

Categorical variables were compared using a Chi-Square test and Fisher’s exact test, whereas quantitative and ordinal variables were compared using a Mann–Whitney U test and Kruskal–Wallis test (with Dune test for multiple comparisons). Spearman’s rho test was used to assess the relationship between biomarkers. Receiver operator characteristic (ROC) curve analyses were performed, and areas under the ROC curves (AUCs) were calculated to evaluate the prognostic accuracy of TK1 expression in discriminating patients with or without distant recurrence and patients who did or did not die due to cancer (Figure A1). Kaplan–Meier method was used to estimate survival outcomes; the log-rank test was used to compare different groups.

All analyses were performed with XLStat (Addinsoft, Paris, FR, version 2021.1.1) and R (R Development Core Team, version 4.1.0), and graphs were made using GraphPad Prism (GraphPad Software, San Diego, CA version 9.1.1). Results were classified as statistically significant if their *p-*values were <0.05.

## 3. Results

### 3.1. Clinicopathological Findings

All patients were females with a mean age at primary BC onset of 52.9 ± 12.6 years (median 51; range 23–85 years). Overall, 56 (70%) cases of BC were pT1 (14 pT1b and 42 pT1c, respectively); 21 (26.25%) were pT2; 2 (2.5%) were pT3; only 1 (1.25%) was pT4.

At the time of the diagnosis: 40 (50%) patients had lymph node metastasis (3 with only micrometastasis and 37 with macrometastasis); the median number of metastatic lymph nodes was 3 (range 0-29). Thirty-five (35) (43.75%) patients had stage I (32, IA and 3, IB, respectively); 25 (31.25%) had stage II (18, IIA and 7, IIB, respectively); 17 (21.25%) had stage III (10, IIIA; 1 IIIB and 6 IIIC, respectively); only 3 (3.75%) had stage IV.

The median maximum tumor diameter was 1.5±1.1 cm. Eighteen (18) patients had multicentric/multifocal (MC/MF) breast cancer (10 with distant metastases and 8 without any recurrence).

Adjuvant chemotherapy ± hormonotherapy had been administrated in 52 (65%) patients. Among patients who experienced distant recurrence, 18 (45%) patients died due to cancer. The loss to follow-up rate was low, with only four patients. The median DRFS (mDRFS) was 97.5 months (range 5–165 months); the median OS (mOS) was 107 months (range 13–206 months). In patients with multifocal/multicentric BC, mDRFS (93.5 months–range 19–129) and mOS (104 months—range 46–136) was slightly lower.

A total of 99 tumor samples were analyzed. Regarding the grade, 24 (24.2%) were G2, and 75 (75.8%) were G3. Ninety-three (93) (93.9%) samples were invasive BC of no special type (NST), 5 (5.1%) were lobular invasive BC, and only 1 (1%) was mucinous BC.

Altogether, 83 (83,8%) samples expressed ER, and 77 (77.8%) also expressed PgR. The median proliferation index was 25% (range 1-90%).

A total of 25 (25.3%) BC samples showed HER2 overexpression or HER2 gene amplification. The surrogate molecular subtyping of the selected cases showed: 29 (29.3%) luminal A (LUM-A), 41 (41.41%) luminal B/HER2- (LUM-B/HER2-), 13 (13.13%) luminal B/HER2+ (LUM-B/HER2+), 12 (12.12%) HER2 overexpressing (HER2-OE), and only 4 (4.04%) triple-negative breast cancers (TNBC).

All clinicopathological findings are summarized in Table 1 and Table 2.

### 3.2. TK1 Expression and Its Association with Clinicopathological Findings and Survival Outcomes

**Figure 1:**
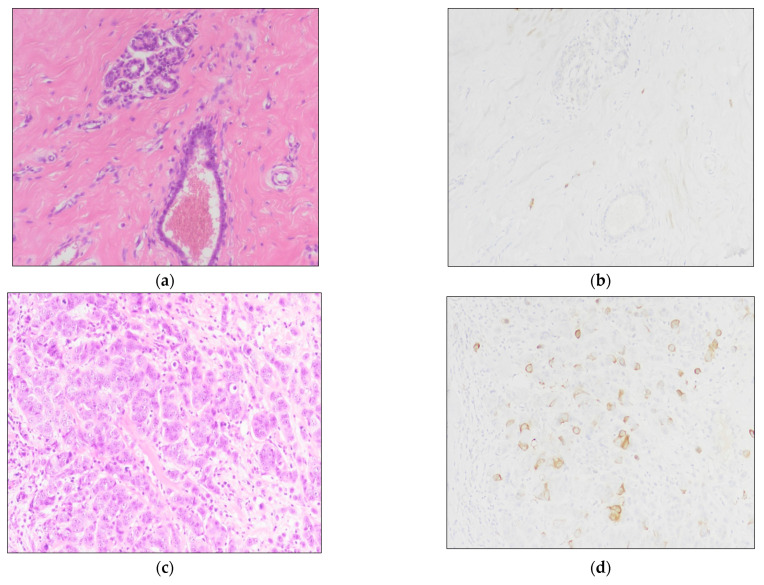
TK1 expression in normal and neoplastic tissue samples: (**a**,**b**) normal breast tissue sample with no expression of TK1; (**b**,**c**) BC tissue sample with moderate to strong cytoplasmic TK1 expression. (**a**,**c**) Hematoxylin and eosin staining. (**b**,**d**) IHC staining for TK1 (see text). Magnification for all images is 200×.

**Figure 2:**
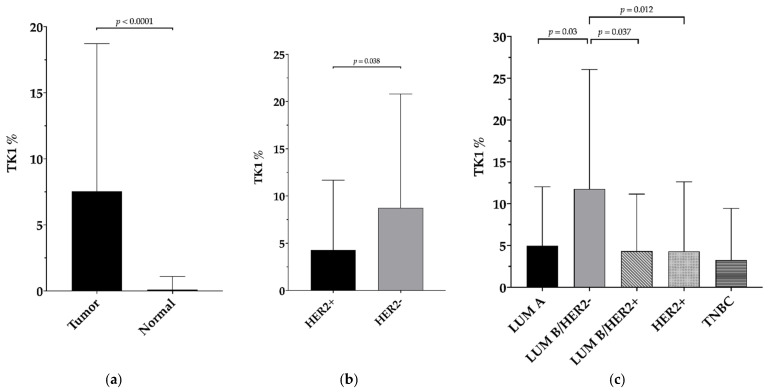
TK1 expression and its association with clinicopathological findings: (**a**) TK1 is significantly overexpressed in BC samples (*p* < 0.0001); (**b**) TK1 expression is lower in BC samples with HER2 overexpression/amplification (*p =* 0.038); (**c**) Luminal B (HER2-) is the BC molecular subtype with the highest TK1 levels (LUM-B/HER2− vs. LUM-A: *p =* 0.003; LUM-B/HER2− vs. LUM-B/HER2+ *p =* 0.037; LUM-B/HER2− vs. HER2+ *p =* 0.012).

TK1 immunohistochemical evaluation is summarized in Table 2 in relation to tumor samples data. TK1 was significantly overexpressed in BC samples compared to normal breast tissue (*p* < 0.0001) (Figure 1 and Figure 2a), and its levels were higher in samples without HER2 overexpression/amplification (*p* = 0.038) (Figure 2b). The levels of TK1 expression were positively correlated with ER (*p <* 0.0001; r_s_ = 0.441) and PgR expression (*p =* 0.04; r_s_ = 0.207). Among the molecular subtypes, Luminal B/HER2− was the group with the highest TK1 expression (LUM-B/HER2− vs. LUM-A: *p =* 0.003; LUM-B/HER2− vs. LUM-B/HER2+ *p =* 0.037; LUM-B/HER2− vs. HER2+ *p =* 0.012). The only group without any statistical difference in respect to LUM-B/HER2− was TNBC (*p =* 0.093), possibly due to the small sample size for this latter category (Figure 2c).

No correlation was found between TK1 expression levels and the other clinicopathological findings (age at diagnosis, tumor grade, invasive cancer size, proliferation index—ki-67, number of metastatic lymph nodes, T, N, M, and stage) and between the two different groups (“metastatic/relapsed” vs. “non-metastatic/relapsed”).

Hereto, TK1 has been validated as a prognostic and predictive biomarker only in serum; therefore, no TK1 cut-off value in BC tissue with prognostic or predictive impact is present in the literature. Hence, due to our small sample size and thanks to the well-balanced groups analyzed (“metastatic/relapsed” vs. “non-metastatic/relapsed”—40 vs. 40), the median value of TK1 expression (2.5%) among all patients was selected as the cut-off to stratify patients’ outcome curves.

To confirm this cut-off value, ROC curve analyses were performed, and AUCs were calculated. The AUC value was 0.57 for the DRFS (*p =* 0.244) and 0.54 for the OS (*p =* 0.642). Though *p*-values were not statistically significant for both ROC curve analyses, the 2.5% cut-off value in the DRFS analysis displayed a sensitivity of 60%, a specificity of 61%, a positive predictive value (PPV) of 61.2%, a negative predictive value (NPV) of 60%, and an accuracy of 60.6%; meanwhile, in the OS analysis, the 2.5% cut-off value revealed a sensitivity of 63.2%, a specificity of 52.6%, a PPV of 25.2%, an NPV of 85.1%, and an accuracy of 54.7%.

**Figure 3:**
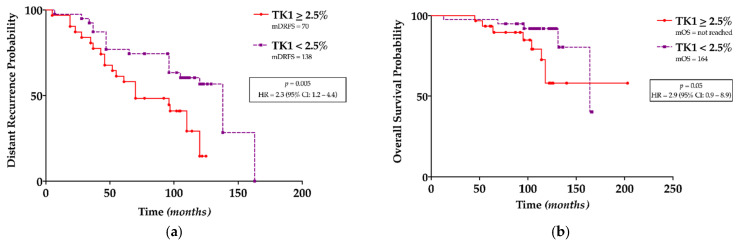
Survival analyses: (**a**) in the cohort undergoing adjuvant chemotherapy, patients with TK1 positive BC (≥2.5%) have a shorter DRFS (mDRFS: 70 vs. 138 months; HR = 2.3, 95% CI 1.2–4.4; *p =* 0.005), (**b**) but not a shorter OS.

In the whole patients’ cohort, TK1 expression > 2.5% was not related to DRFS (Figure S1a) or OS (Figure S1b). However, in the cohort of patients undergoing adjuvant chemotherapy, TK1 > 2.5% was strongly associated with a shorter DRFS (mDRFS: 70 vs. 138 months; HR=2.3, 95% CI 1.2–4.4; *p =* 0.005) (Figure 3a) but not with OS (Figure 3b). No correlation was found in the cohort of patients with only surgically treated BC (Figure S1c,d). Even stratifying patients according to their BC molecular subtype, TK1 expression was not associated with DRFS or OS (Figure S2a–f).

The ki-67 cut-off values of 20 ± 5%, generally used (together with other clinicopathological parameters) to drive medical, therapeutic decisions, did not allow a prognostic stratification of our cohort, since it was not correlated with DRFS or OS (Figure S3a–f).

## 4. Discussion

Breast cancer (BC), the most common non-cutaneous malignant tumor in women, is highly heterogeneous in terms of clinical course and outcome. Despite all adjuvant therapies, approximately one in four patients with early stage BC experiences recurrence with distant metastases [26]. This may be related to inefficient chemotherapeutic approaches due to the not yet fully understood cancer biology and/or by the insufficient stratification of patients.

BC is classified into four major molecularly defined subtypes: luminal A, luminal B, HER2+, and triple-negative (TN) tumors; prognosis and pattern of metastasis vary among these subgroups [27]. Although most relapses occur during the first 5 years after diagnosis, late recurrence has been reported, especially in luminal breast cancers [28]. Thus, the identification of predictors of relapse later than 5-10 years in BC represents an unmet clinical need. Predicting patients with a high risk of late relapse may indeed change individual follow-up management and define long-term hormonal therapy in the right patients [29]. In the present study, we therefore focused our analysis on luminal BC tumors, exploring more patients with this specific subtype.

Among the large variety of possible candidates as predictive biomarkers to explore, TK1 has been of particular interest for us since it has been recently shown that high TK1 activity and expression levels in the serum of BC patients are associated with a higher risk of recurrence [30,31], PFS, and OS [32], and that its overexpression in plasma-derived exosomes is associated with clinical resistance to CDK4/6 inhibitors in patients with metastatic BC [33].

Cytosolic thymidine kinase 1 (TK1) is a well-known cell cycle-regulated enzyme crucial for nucleotide metabolism during DNA synthesis [34]. TK1 is involved in the nucleotide salvage pathway and catalyzes the conversion of thymidine to deoxythymidine monophosphate, which is further phosphorylated to di- and triphosphates preceding its incorporation into DNA [8]. The activity of TK1 is low or absent in resting cells, increasing in the G1/S transition, peaking in the S phase, and then disappearing during mitosis [35].

The molecular mechanisms of cancer progression and metastasization involving TK1 have only been recently demonstrated. In particular, in in vitro models of lung and breast cancers, dTTP produced by TK1 was demonstrated to allosterically activate the enzyme Ribonucleotides Reductase (RNR), which converts GDP to deoxiGDP. The imbalance between GTP and GDP, in turn, activates GTPase proteins such as RhoA, which then stimulates tumor proliferation and progression. Moreover, loss of TK1 results in a loss of GDF15, one of the TGF-beta ligands, leading to reduced tumor activity and metastasization [36]. Interestingly, He et al. have also suggested how TK1 may represent a more reliable proliferation biomarker than ki-67 in lung, colorectal, and breast cancers; in the latter, TK1 is better discriminated between breast cancer stages [37].

In this study, TK1 expression was assessed by immunohistochemistry in a cohort of 80 BC patients with different distant recurrence rates (between 5 and 165 months) and long-term follow-up (between 13 and 206 months). We correlated TK1 expression with clinicopathological characteristics of the tumors and survival outcomes, with the aim to better understand the role of TK1 in BC biology and to suggest it as a useful biomarker in the stratification of BC patients’ prognosis.

Interestingly, TK1 positively correlated with hormonal receptors (ER and PgR) and was more expressed in tumors without HER2 overexpression/amplification. These results support recent data from the literature showing TK1 as a potential pharmacodynamic marker of CDK4/6 inhibition in ER+/HER2- BC [38]; indeed, TK1 synthesis is regulated by the E2F pathway [39], which represents the main target of CDK4/6 inhibitors. So far, little has been published on the association of TK1 with clinicopathological parameters in BC patients: Nisman et al., in 2010, reported a correlation of serum TK1 and advanced T stage, higher grade, presence of tumor necrosis, vascular invasion, and lack of hormonal receptors expression [16]. Surprisingly, these data are, at least partially, in contrast with our results where TK1 positively correlated with ER/PgR expression; however, this should be related to a selection bias. In our cohort, luminal B is the most frequent molecular subtype (54.54%). This overrepresentation is due to the exclusion of neoadjuvant-treated patients. Indeed, according to clinical practice guidelines, neoadjuvant chemotherapy represents the standard of care for HER2+ and triple-negative BC nowadays [40]; consequently, these two latter subtypes are less represented among our patients (12.12% and 4.04%, respectively). Finally, discrepancies between tissue and serum levels of biomarkers may occur [41] and should represent, in our opinion, a boost for future studies on the biology of cancer and the monitoring of cancer patients.

According to our results, TK1 is most highly expressed in luminal B/HER2- tumors that are characterized by a more aggressive behavior than luminal A tumors and frequently display late relapse despite adjuvant therapies [42]. This strongly supported our hypothesis of a possible role of TK1 in the stratification of BC prognosis. In order to analyze this intriguing issue, we applied the value of TK1 found with ROC analysis among all patients (2.5% of cytoplasmic immunohistochemical expression) as the cut-off to stratify patients’ outcomes. Surprisingly, among patients undergoing adjuvant chemotherapy and thus affected by a more aggressive disease, lower TK1 levels (<2.5%) correlated with better DRFS. So far, TK1 has been validated as a prognostic and predictive biomarker in BC patients only in serum; therefore, to the best of our knowledge, no tissue TK1 cut-off value with prognostic or predictive impact is present in the literature. According to these results, a cut-off value of 2.5% may represent a potentially useful tool in the stratification of cancer prognosis among patients affected by luminal B/HER2- tumors and undergoing adjuvant chemotherapy. These data may pave the way for novel strategies in the treatment and monitoring of BC patients.

## 5. Potential Pitfalls

The major strengths of the current study are: (i) it represents one of the most well-characterized selections of BC cases on this subject; (ii) patients were clinically followed over a long period of time. On the other hand, the present study also has several limitations: (i) its retrospective nature; (ii) the relatively small sample size due to the rigid criteria used for samples collection: to overcome this limitation, these preliminary data need to be confirmed on a larger multi-institutional cohort or with an external validation cohort of patients; (iii) a low cut-off point of TK1 expression, such as the 2.5% proposed herein, may be difficult to calculate by a trained pathologist through a microscope eyepiece: in order to overcome this issue, some may consider digital imaging as a potential tool of benefit to the pathology department in the routine evaluation of novel biomarkers for the stratification of cancer-related risk [43].

## 6. Conclusions

Breast cancer (BC) is characterized by a highly heterogeneous clinical course and outcome. Despite remarkable efforts, the full elucidation of its biology has not yet been reached. Novel therapeutic approaches improved patients’ outcomes; however, approximately one in four patients experience delayed distant recurrence. The delayed metastasis prediction value of several clinicopathological and molecular BC features have been investigated so far, but with inconsistent or incomplete results. Thymidine kinase-1 (TK1) turned out to be an important serum biomarker with a prognostic and predictive value in BC patients. To our knowledge, this is the first translational research study that tries to use TK1 expression in the clinical routine for the prognostic stratification of BC patients.

Our preliminary data suggest that patients with LUM B/HER2-/TK1+ BC undergoing adjuvant chemotherapy may require a longer and closer follow-up after surgery and/or a different adjuvant therapeutic algorithm.

## Figures and Tables

**Table 1:** Clinicopathological characteristics of patients.

**Patients**	80
**Age** *(range)*	52.9 (23–85)
**pT**	**n. (%)**
*pT1b*	14 (17.5)
*pT1c*	42 (52.5)
*pT2*	21 (26.25)
*pT3*	2 (2.5)
*pT4*	1 (1.25)
**pN**	
*pN0*	40 (50)
*pN1mi*	3 (3.75)
*pN1*	21 (26.25)
*pN2*	9 (11.25)
*pN3*	7 (8.75)
**M**	
*M0*	77 (96.25)
*M1*	3 (3.75)
**Stage** *(AJCC 2017)*	
*IA*	32 (40)
*IB*	3 (3.75)
*IIA*	18 (22.5)
*IIB*	7 (8.75)
*IIIA*	10 (12.5)
*IIIB*	1 (1.25)
*IIIC*	6 (7.5)
*IV*	3 (3.75)
**Multicentric/Multifocal**	
*Yes*	18 (22.5)
*No*	62 (77.5)
**Adjuvant Chemotherapy**	
*Yes*	52 (65)
*No*	28 (35)
**Distant Recurrence**	
*Yes*	40 (50)
*No*	40 (50)
*mDRFS (months—range)*	97.5 (5–165)
**Cancer-Related Death**	
*Yes*	18 (22.5)
*No*	62 (77.5)
*mOS (months—range)*	107 (13–206)

**Table 2:** Tumor samples’ features.

**Tumor Samples**		99	**TK1 Positivity**
**Grade** *(Nottingham combined histologic grade)*		**n. (%)**	**Median (range)**
*G2*		24 (24.2)	2.50 (0–25)
*G3*		75 (75.8)	3.30 (0–60)
**Histotype** *(WHO 2019)*			
*NST*		93 (93.94)	2.50 (0–60)
*Lobular*		5 (5.05)	2.00 (0–28.33)
*Mucinous*		1 (1.01)	16.67 (*)
**Hormone Receptor Status**			
*ER*	*Positive*	83 (83.8)	3.30 (0–60)
	*Negative*	16 (16.2)	0.33 (0–25)
*PgR*	*Positive*	78 (78.8)	3.30 (0–60)
	*Negative*	21 (21.2)	0.46 (0–28.33)
**HER2 status**			
*Amplified*		25 (25.3)	0.67 (0–25)
*Not amplified*		74 (74.7)	3.30 (0–60)
**Molecular subtype**			
*LUM-A*		29 (29.3)	1.67 (0–28.33)
*LUM-B/HER2-*		41 (41.41)	5.00 (0–60)
*LUM-B/HER2+*		13 (13.13)	2.00 (0–25)
*HER2-OE*		12 (12.12)	0.33 (0–18.33)
*Triple-negative*		4 (4.04)	0.33 (0–12.50)

(*) Since in our cohort, one tumor sample was mucinous, its TK1 positivity has been reported as a single value.

## Supplementary Materials

The following are available online at https://www.mdpi.com/article/10.3390/jcm10225416/s1, Figure S1: survival analyses in the whole cohort and in surgically treated patients only. Figure S2: survival analyses according the BC molecular stratification. Figure S3: survival analyses according to different ki-67 cut-off points.

## References

[1] Sung H., Ferlay J., Siegel R.L., Laversanne M., Soerjomataram I., Jemal A., Bray F. (2021). Global cancer statistics 2020: Globocan estimates of incidence and mortality worldwide for 36 cancers in 185 countries. CA Cancer J. Clin..

[2] Kim Y., Yoo K.Y., Goodman M.T. (2015). Differences in incidence, mortality and survival of breast cancer by regions and countries in asia and contributing factors. Asian Pac. J. Cancer Prev..

[3] Johansson I., Killander F., Linderholm B., Hedenfalk I. (2014). Molecular profiling of male breast cancer—Lost in translation?. Int. J. Biochem. Cell Biol..

[4] Fanelli G.N., Naccarato A.G., Scatena C. (2020). Recent advances in cancer plasticity: Cellular mechanisms, surveillance strategies, and therapeutic optimization. Front. Oncol..

[5] Lehmann B.D., Bauer J.A., Chen X., Sanders M.E., Chakravarthy A.B., Shyr Y., Pietenpol J.A. (2011). Identification of human triple-negative breast cancer subtypes and preclinical models for selection of targeted therapies. J. Clin. Investig..

[6] Van Mechelen M., Van Herck A., Punie K., Nevelsteen I., Smeets A., Neven P., Weltens C., Han S., Vanderstichele A., Floris G. (2020). Behavior of metastatic breast cancer according to subtype. Breast Cancer Res. Treat..

[7] Chen Y.L., Eriksson S., Chang Z.F. (2010). Regulation and functional contribution of thymidine kinase 1 in repair of DNA damage. J. Biol. Chem..

[8] Bitter E.E., Townsend M.H., Erickson R., Allen C., O’Neill K.L. (2020). Thymidine kinase 1 through the ages: A comprehensive review. Cell Biosci..

[9] Xu Y., Liu B., Shi Q.L., Huang P.L., Zhou X.J., Ma H.H., Lu Z.F., Bo Y., Eriksson S., He E. (2014). Thymidine kinase 1 is a better prognostic marker than ki-67 for pt1 adenocarcinoma of the lung. Int. J. Clin. Exp. Med..

[10] O’Neill K.L., Buckwalter M.R., Murray B.K. (2001). Thymidine kinase: Diagnostic and prognostic potential. Expert Rev. Mol. Diagn..

[11] Topolcan O., Holubec L. (2008). The role of thymidine kinase in cancer diseases. Expert Opin. Med. Diagn..

[12] Zhou J., He E., Skog S. (2013). The proliferation marker thymidine kinase 1 in clinical use. Mol. Clin. Oncol..

[13] Li L.T., Jiang G., Chen Q., Zheng J.N. (2015). Ki67 is a promising molecular target in the diagnosis of cancer (review). Mol. Med. Rep..

[14] Alegre M.M., Robison R.A., O’Neill K.L. (2012). Thymidine kinase 1 upregulation is an early event in breast tumor formation. J. Oncol..

[15] Guan H., Sun Y., Zan Q., Xu M., Li Y., Zhou J., He E., Eriksson S., Wen W., Skog S. (2009). Thymidine kinase 1 expression in atypical ductal hyperplasia significantly differs from usual ductal hyperplasia and ductal carcinoma in situ: A useful tool in tumor therapy management. Mol. Med. Rep..

[16] Nisman B., Allweis T., Kaduri L., Maly B., Gronowitz S., Hamburger T., Peretz T. (2010). Serum thymidine kinase 1 activity in breast cancer. Cancer Biomark..

[17] He E., Xu X.H., Guan H., Chen Y., Chen Z.H., Pan Z.L., Tang L.L., Hu G.Z., Li Y., Zhang M. (2010). Thymidine kinase 1 is a potential marker for prognosis and monitoring the response to treatment of patients with breast, lung, and esophageal cancer and non-hodgkin’s lymphoma. Nucleosides Nucleotides Nucleic Acids.

[18] World Health Organization, International Agency for Research on Cancer (2019). Breast Tumours.

[19] American Joint Committee on Cancer (2017). AJCC Cancer Staging Manual.

[20] Elston C.W., Ellis I.O. (1991). Pathological prognostic factors in breast cancer. I. The value of histological grade in breast cancer: Experience from a large study with long-term follow-up. Histopathology.

[21] Allison K.H., Hammond M.E.H., Dowsett M., McKernin S.E., Carey L.A., Fitzgibbons P.L., Hayes D.F., Lakhani S.R., Chavez-MacGregor M., Perlmutter J. (2020). Estrogen and progesterone receptor testing in breast cancer: American society of clinical oncology/college of american pathologists guideline update. Arch. Pathol. Lab. Med..

[22] Wolff A.C., Hammond M.E.H., Allison K.H., Harvey B.E., Mangu P.B., Bartlett J.M.S., Bilous M., Ellis I.O., Fitzgibbons P., Hanna W. (2018). Human epidermal growth factor receptor 2 testing in breast cancer: American society of clinical oncology/college of american pathologists clinical practice guideline focused update. Arch. Pathol. Lab. Med..

[23] Nielsen T.O., Leung S.C.Y., Rimm D.L., Dodson A., Acs B., Badve S., Denkert C., Ellis M.J., Fineberg S., Flowers M. (2021). Assessment of ki67 in breast cancer: Updated recommendations from the international ki67 in breast cancer working group. J. Natl. Cancer Inst..

[24] Howlader N., Cronin K.A., Kurian A.W., Andridge R. (2018). Differences in breast cancer survival by molecular subtypes in the united states. Cancer Epidemiol. Biomark. Prev..

[25] Wolff A.C., Hammond M.E., Hicks D.G., Dowsett M., McShane L.M., Allison K.H., Allred D.C., Bartlett J.M., Bilous M., Fitzgibbons P. (2013). Recommendations for human epidermal growth factor receptor 2 testing in breast cancer: American society of clinical oncology/college of american pathologists clinical practice guideline update. J. Clin. Oncol..

[26] Early Breast Cancer Trialists’ Collaborative Group (2012). Comparisons between different polychemotherapy regimens for early breast cancer: Meta-analyses of long-term outcome among 100,000 women in 123 randomised trials. Lancet.

[27] Kaplan M.A., Arslan U.Y., Isikdogan A., Dane F., Oksuzoglu B., Inanc M., Akman T., Kucukoner M., Cinkir H.Y., Rzazade R. (2016). Biological subtypes and distant relapse pattern in breast cancer patients after curative surgery (study of anatolian society of medical oncology). Breast Care.

[28] Lim E., Metzger-Filho O., Winer E.P. (2012). The natural history of hormone receptor-positive breast cancer. Oncology.

[29] Davies C., Pan H., Godwin J., Gray R., Arriagada R., Raina V., Abraham M., Medeiros Alencar V.H., Badran A., Bonfill X. (2013). Long-term effects of continuing adjuvant tamoxifen to 10 years versus stopping at 5 years after diagnosis of oestrogen receptor-positive breast cancer: Atlas, a randomised trial. Lancet.

[30] He Q., Fornander T., Johansson H., Johansson U., Hu G.Z., Rutqvist L.E., Skog S. (2006). Thymidine kinase 1 in serum predicts increased risk of distant or loco-regional recurrence following surgery in patients with early breast cancer. Anticancer Res..

[31] Nisman B., Allweis T., Kadouri L., Mali B., Hamburger T., Baras M., Gronowitz S., Peretz T. (2013). Comparison of diagnostic and prognostic performance of two assays measuring thymidine kinase 1 activity in serum of breast cancer patients. Clin. Chem. Lab. Med..

[32] Bjohle J., Bergqvist J., Gronowitz J.S., Johansson H., Carlsson L., Einbeigi Z., Linderholm B., Loman N., Malmberg M., Soderberg M. (2013). Serum thymidine kinase activity compared with ca 15-3 in locally advanced and metastatic breast cancer within a randomized trial. Breast Cancer Res. Treat..

[33] Del Re M., Bertolini I., Crucitta S., Fontanelli L., Rofi E., De Angelis C., Diodati L., Cavallero D., Gianfilippo G., Salvadori B. (2019). Overexpression of tk1 and cdk9 in plasma-derived exosomes is associated with clinical resistance to cdk4/6 inhibitors in metastatic breast cancer patients. Breast Cancer Res. Treat..

[34] Welin M., Kosinska U., Mikkelsen N.E., Carnrot C., Zhu C., Wang L., Eriksson S., Munch-Petersen B., Eklund H. (2004). Structures of thymidine kinase 1 of human and mycoplasmic origin. Proc. Natl. Acad. Sci. USA.

[35] Bagegni N., Thomas S., Liu N., Luo J., Hoog J., Northfelt D.W., Goetz M.P., Forero A., Bergqvist M., Karen J. (2017). Serum thymidine kinase 1 activity as a pharmacodynamic marker of cyclin-dependent kinase 4/6 inhibition in patients with early-stage breast cancer receiving neoadjuvant palbociclib. Breast Cancer Res..

[36] Malvi P., Janostiak R., Nagarajan A., Cai G., Wajapeyee N. (2019). Loss of thymidine kinase 1 inhibits lung cancer growth and metastatic attributes by reducing gdf15 expression. PLoS Genet..

[37] He Q., Mao Y., Wu J., Decker C., Merza M., Wang N., Eriksson S., Castro J., Skog S. (2004). Cytosolic thymidine kinase is a specific histopathologic tumour marker for breast carcinomas. Int. J. Oncol..

[38] McCartney A., Bonechi M., De Luca F., Biagioni C., Curigliano G., Moretti E., Minisini A.M., Bergqvist M., Benelli M., Migliaccio I. (2020). Plasma thymidine kinase activity as a biomarker in patients with luminal metastatic breast cancer treated with palbociclib within the trend trial. Clin. Cancer Res..

[39] Bello L.J. (1974). Regulation of thymidine kinase synthesis in human cells. Exp. Cell. Res..

[40] Sharma P., Connolly R.M., Roussos Torres E.T., Thompson A. (2020). Best foot forward: Neoadjuvant systemic therapy as standard of care in triple-negative and HER2-positive breast cancer. Am. Soc. Clin. Oncol. Educ. Book.

[41] Rodriguez J., Avila J., Rolfo C., Ruiz-Patino A., Russo A., Ricaurte L., Ordonez-Reyes C., Arrieta O., Zatarain-Barron Z.L., Recondo G. (2021). When tissue is an issue the liquid biopsy is nonissue: A review. Oncol. Ther..

[42] Tran B., Bedard P.L. (2011). Luminal-b breast cancer and novel therapeutic targets. Breast Cancer Res..

[43] Klein C., Zeng Q., Arbaretaz F., Devevre E., Calderaro J., Lomenie N., Maiuri M.C. (2021). Artificial intelligence for solid tumor diagnosis in digital pathology. Br. J. Pharmacol..

