# Supplementary material for: Immunohistochemistry for Thymidine Kinase-1 (TK1): A Potential Tool for the Prognostic Stratification of Breast Cancer Patients
Source: J Clin Med. 2021 Nov 19;10(22):5416. doi: 10.3390/jcm10225416 (PMC8623797; doi:10.3390/jcm10225416)
Supplement: Supplementary file 1 [file jcm-10-05416-s001.zip › jcm-1455263-supplementary.pdf]

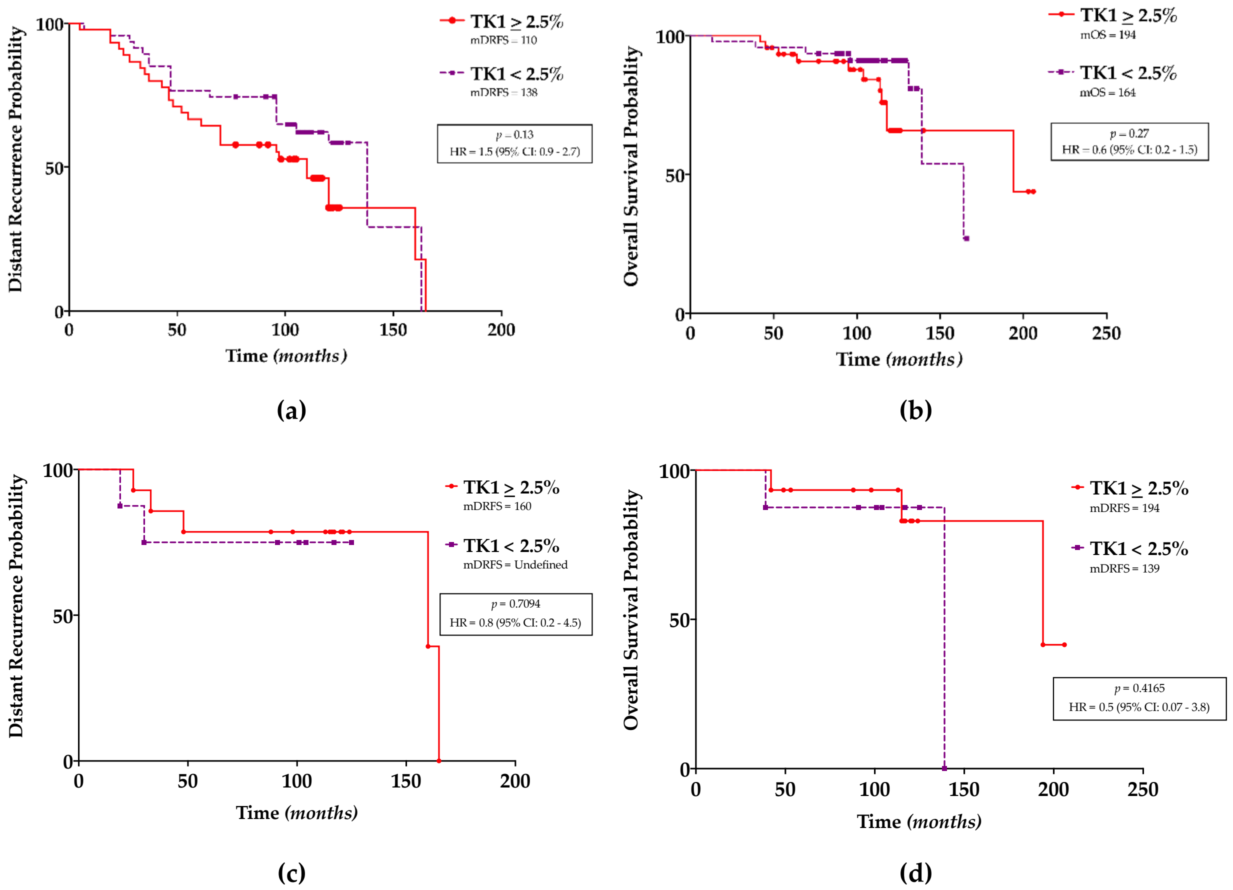

Figure S1. Survival analyses: **(a)** in the whole cohort, TK1 is not related to DRFS, **(b)** or OS; **(c)** in the cohort of only surgically treated patients, TK1-positive (≥2.5 %) BC patients do not have a shorter DRFS **(d)** or OS.

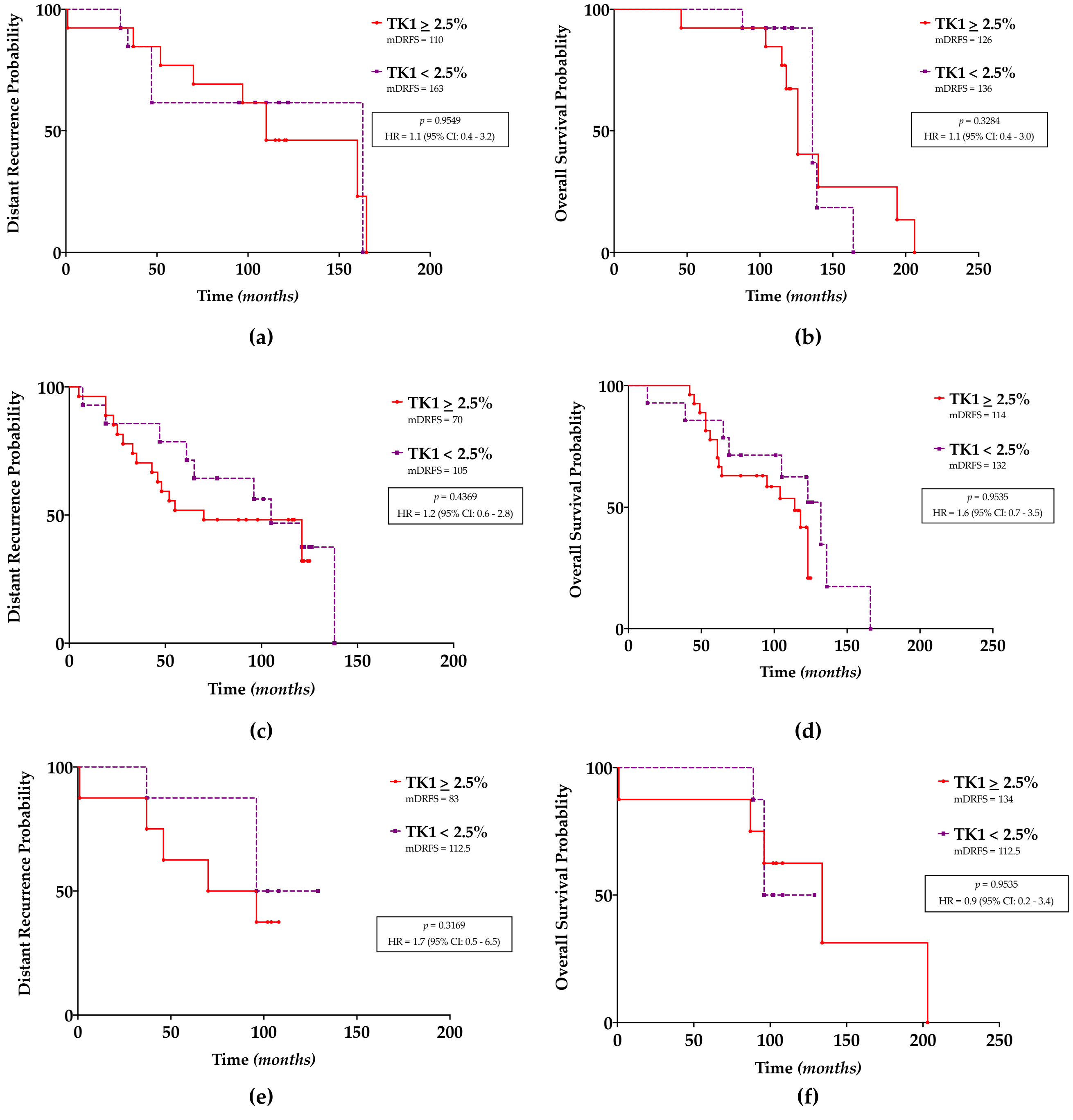

Figure S2. Survival analyses according to BC molecular stratification: in any of the luminal subtype samples TK1 is not related to DRFS (**a: LUM-A, c: LUM-B/HER2-, e: LUM-B/HER2+**), or OS **(b: LUM-A, d: LUM-B/HER2-, f: LUM-B/HER2+)**. Survival analyses in HER2-OE BC (12 samples) and TNBC (4 samples) were not performed given the limited number of comparisons.

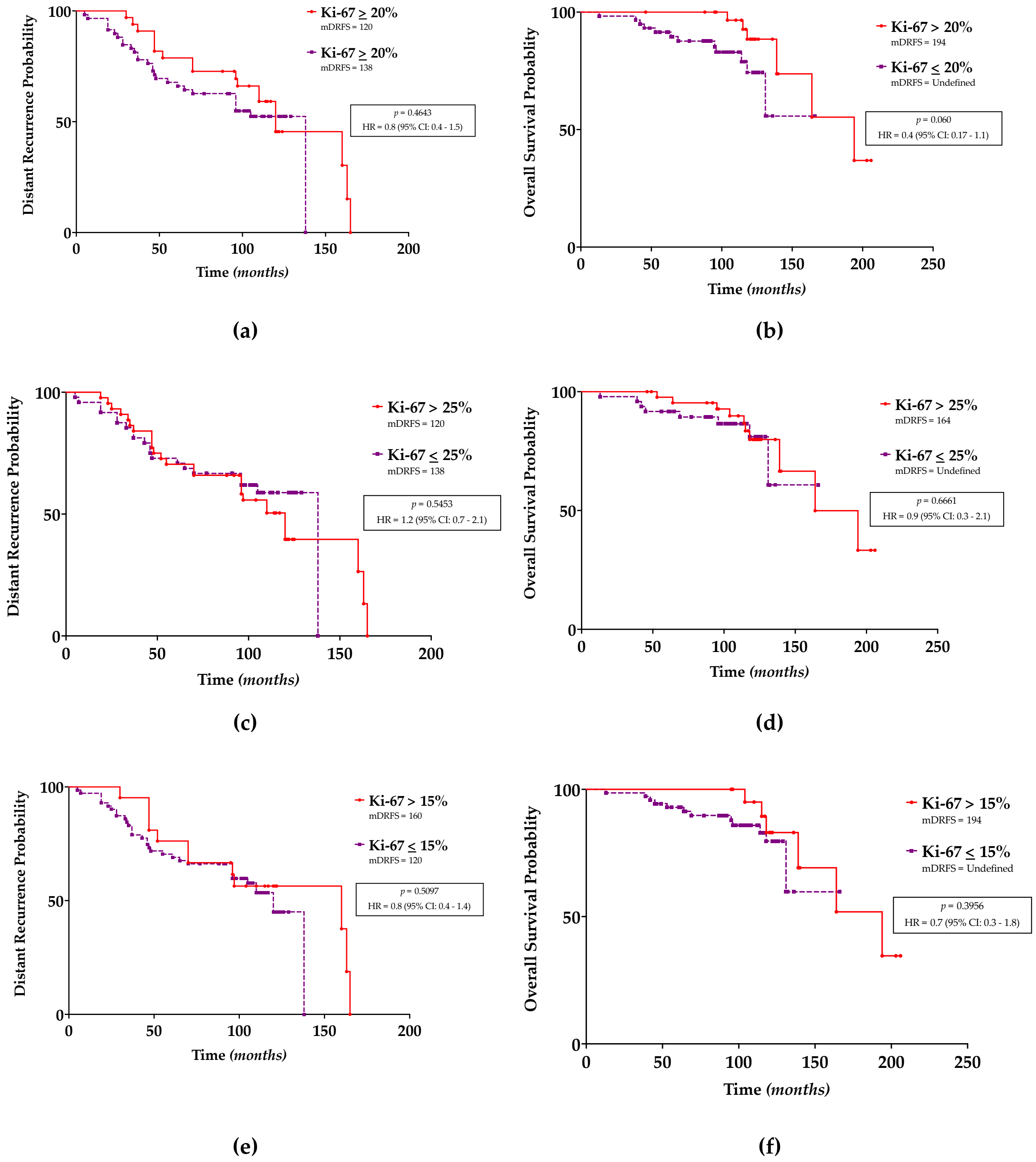

Figure S3. Survival analyses according to Ki-67: none of the ki-67 cut-off values used (**a, c, e**) were related to DRFS or **(b, d, f)** OS.
